# Postpartum hemorrhage prevention in Nepal: a program assessment

**DOI:** 10.1186/s12884-017-1347-z

**Published:** 2017-06-05

**Authors:** Swaraj Pradhan Rajbhandari, Kamal Aryal, Wendy R. Sheldon, Bharat Ban, Senendra Raj Upreti, Kiran Regmi, Shilu Aryal, Beverly Winikoff

**Affiliations:** 1 Gynuity Health Projects, 15 East 26th Street, Suite 801, New York, NY 10010 USA; 2 New ERA (P) Ltd., Kathmandu, Nepal; 3 Family Health Division, Department of Health Services, Ministry of Health and Population Kathmandu, Kathmandu, Nepal

**Keywords:** Postpartum hemorrhage, Misoprostol, Advance distribution, Home birth, Nepal

## Abstract

**Background:**

In 2009, the Nepal Ministry of Health and Population launched a national program for prevention of postpartum hemorrhage (PPH) during home births that features advance distribution of misoprostol to pregnant women. In the years since, the government has scaled-up the program throughout much of the country. This paper presents findings from the first large-scale assessment of the effectiveness of the advance distribution program.

**Methods:**

Data collection was carried out in nine districts and all three ecological zones. To assess knowledge, receipt and use of misoprostol, household interviews were conducted with 2070 women who had given birth within the past 12 months. To assess supply and provision of misoprostol, interviews were conducted with 270 Female Community Health Volunteers (FCHVs) and staff at 99 health facilities.

**Results:**

Among recently delivered women, only 15% received information about misoprostol and 13% received misoprostol tablets in advance of delivery. Yet 87% who received advance misoprostol and delivered at home used it for PPH prevention. Among FCHVs, 96% were providing advance misoprostol for PPH prevention; however 81% had experienced at least one misoprostol stock out within the past year. About one-half of FCHVs were providing incomplete information about the use of misoprostol; in addition, many did not discuss side effects, how to recognize PPH or where to go if PPH occurs. Among health facilities, just one-half had sufficient misoprostol stock, while 95% had sufficient oxytocin stock, at the time of this assessment.

**Conclusions:**

In Nepal, women who receive advance misoprostol are both willing and able to use the medication for PPH prevention during home births. However the supply and personnel challenges identified raise questions about scalability and impact of the program over the long-term. Further assessment is needed.

## Background

The government of Nepal has made considerable progress in its efforts to promote safe motherhood. For instance, between 2006 and 2011 there was a 36% increase in the provision of antenatal care (from 44 to 60% of all pregnant women); and a 94% increase in the rate of facility-based births (from 18 to 35% of all births) [1, 2]. However, an estimated two-thirds of women still give birth at home with no skilled birth attendant present and maternal mortality, despite recent declines, remains high. The most recent maternal mortality ratio was estimated to be 281 per 100,000 live births [1], about 25% of which has been attributed to postpartum hemorrhage (PPH) [3, 4].

### Postpartum hemorrhage (PPH) prevention program

In recent years, the government of Nepal has invested in implementing and scaling-up its PPH prevention program throughout much of the country. The main goal is to ensure uterotonic coverage immediately following birth of the baby at all deliveries, regardless of delivery location. The program is run by the government’s Family Health Division (FHD) within the Department of Health Services, in partnership with multiple organizations. It is governed by a set of guidelines that includes a detailed plan for program implementation [5]. The PPH prevention program consists of two main components. The first entails provision of oxytocin during facility-based births. In particular, skilled birth attendants are instructed to provide 10 International Units (IU) of injectable oxytocin immediately post-partum to all women who deliver in government health facilities. Oxytocin is widely available in facilities throughout the country [6] and has been the standard treatment for active management of the third stage of labor for many years.

The second component of the PPH prevention program involves advance distribution of misoprostol to pregnant women for use during home births. Following the success of a 2005 pilot project involving advance distribution of misoprostol to pregnant women in the rural areas of Banke district, the Nepal government began planning for a country-wide program for all home births [7, 8] The ‘Matri Surakhchya Chakki’(MSC) or “advance distribution” program was launched in 2009 and at the time of this assessment, had been implemented in 27 of the country’s 75 districts. The program is carried out by Female Community Health Volunteers (FCHVs) who identify pregnant women in their communities and encourage them to visit their local health facility for antenatal care at around 4 months of pregnancy. FCHVs also distribute iron, folic acid and anti-worm tablets to pregnant women in their communities and, during the eighth month of pregnancy, they provide 600 mcg misoprostol (in the form of three 200 mcg tablets), along with counseling on proper usage of the pills for prevention of PPH during home birth. The FCHVs instruct women to take all three pills immediately following birth of the baby, and prior to placental delivery. Until recently, the women were also instructed to return any unused medication after the birth.

For the advance distribution component, the FHD provides initial training to all new FCHVs and includes material devoted to PPH and the provision of misoprostol. The FHD also provides supervision and refresher training to the FCHVs, although the advance distribution program is not always covered in these trainings.

### Current study

In order to examine the effectiveness of the national PPH prevention program, a study was carried out in 2013 by the Family Health Division, Gynuity Health Projects and New ERA. Although the primary focus was the advance distribution of misoprostol, some aspects of the facility-based provision of oxytocin were also examined. The primary study objectives were to assess the availability, provision and use of misoprostol for home births. This article summarizes the key findings, including the frequency of misoprostol receipt and use among home births; stock of misoprostol and prevalence of program-related knowledge and practices among FCHVs; and supply of uterotonics at government health facilities.

## Methods

To assess knowledge, receipt and use of advance misoprostol, household interviews were conducted with 2070 women who had given birth within the past 12 months (recently delivered women) (690 per each of the country’s three ecological regions comprised of mountain, hill and terai/flatland terrain). Trained interviewers approached a total of 21,767 households to screen for eligibility. If the woman was deemed eligible and gave verbal consent to participate, the interviewer asked a series of questions about demographic characteristics; use of antenatal and delivery services; and knowledge, receipt and use of misoprostol for PPH prevention. Each interview took approximately 20 min to complete.

To assess the supply and provision of misoprostol, interviews were conducted with 270 FCHVs (90 per ecological region) and staff at 99 government health facilities (33 per ecological region). Trained interviewers asked FCHVs about their knowledge of misoprostol for PPH prevention, current and recent misoprostol stock, program training and supervision, and how to improve the PPH prevention program. Trained interviewers also administered surveys to the focal person of the PPH prevention program within each health facility. Survey items included questions about PPH prevention practices and drugs; availability, storage and supply of uterotonics; and suggestions for improving the PPH prevention program.

Data were collected between May 2013 and August 2013 by 30 female interviewers and 10 field supervisors. For the household interviews, the field staff was divided into ten teams, each comprised of three female interviewers and one supervisor. The interviews with FCHVs and the health facility staff were conducted by the field supervisors. All field staff received a one-week training prior to the start of field mobilization. Review of completed data collection forms and data entry were carried out by New ERA in Nepal.

The primary variable of interest was advance receipt of misoprostol for PPH prevention among recently delivered women. Receipt was defined as having received advance misoprostol from an FCHV or other health provider for the purpose of PPH prevention during a home birth. Using evidence from previous district-level studies, we hypothesized that receipt of advance misoprostol would differ by ecological region, with prevalence of 37% in the mountain region, 47% in the hill region, and 57% in the terai region [9–11]. We calculated an adjusted alpha for all three ecological regions using Bonferroni correction and assuming an inter-group correlation of 0.50. The sample requirements for binomial differences in proportions were calculated using α = 0.0289 and β = 0.80 for all groups. Since we desired equal sample sizes, we chose the highest sample requirement. We assumed a design effect of 1.50, resulting in a final minimum sample requirement of 2052 (684 per ecological region).

Selection of the household interview sample was carried out using a three-stage cluster design. In the first stage, nine districts were selected purposively from the 27 districts in which the advance distribution program had been fully implemented at the time of the evaluation, in order to ensure fair representation of all ecological and development regions. Three districts were chosen from each of the country’s three ecological regions (mountain, hill and terai). Next, 30 rural clusters were selected from among the three sample districts in each ecological region using probability proportional to size (PPS) methodology. Only rural clusters were considered since rural areas are the key focus of the advance distribution program, given that home births are highest in rural areas. Finally, within each rural cluster, households were screened until a total of 23 interviews had been completed.

Within each rural cluster, the health facility located in the rural municipality (also known as the village development committee) was included in the facility sample. In addition, the district hospital from each sample district was included. This provided a total of 99 facilities (33 per ecological region). A total of three FCHVs per rural cluster were also recruited for interviews (or if there were more than three FCHVs in a cluster, three were randomly selected). This provided a total of 270 FCHVs (90 per ecological region).

### Statistical analysis

We used frequencies to examine program-related variables and background characteristics among recently delivered women, FCHVs, and health facilities. We used Pearson’s Chi-Square and Fisher’s Exact tests for categorical variables to assess regional differences in receipt and use of advance misoprostol among recently delivered women. The analysis was conducted using Stata/SE 12.1 and *P*-values <0.05 were considered significant.

## Results

Table 1 summarizes the background, antenatal and birth characteristics of the household interview respondents (recently delivered women). Educational attainment and wealth quintiles were each lowest in the mountain region and highest in the terai region. Receipt of antenatal care was universally high and so was the use of iron/folic acid during pregnancy. Health providers (doctors, nurses or auxiliary nurse midwives) were the primary source of antenatal care although about one-third of women also received some antenatal care from FCHVs. Overall, about two-thirds of recently delivered women met with an FCHV during pregnancy. More than half gave birth in a health facility although there was regional variation, with the prevalence of facility births highest in the terai region and lowest in the mountain region.

**Table 1:** Recently delivered women: Background, antenatal and birth characteristics

	Mountain region	Hill region	Terai region
	(*n* = 690)	(*n* = 690)	(*n* = 690)
Mean age in years ± SD	24 ± 6	25 ± 6	23 ± 5.0
Educational attainment			
None	50.4 (348)	39.4 (272)	29.6 (204)
Primary	36.2 (250)	42.9 (296)	57.1 (394)
Secondary plus	13.3 (92)	17.7 (122)	13.3 (92)
Wealth quintile			
Lowest	45.2 (312)	14.8 (102)	0.0 (0)
Second	36.7 (253)	22.8 (157)	0.1 (1)
Third	12.2 (84)	36.2 (250)	12.0 (83)
Fourth	1.6 (11)	15.1 (104)	43.3 (299)
Highest	4.4 (30)	11.2 (77)	44.5 (307)
Met with FCHV during pregnancy	61.9 (427)	60.1 (415)	78.0 (538)
Received antenatal care	89.3 (616)	90.9 (627)	94.6 (653)
Source of antenatal care^a^	*n = 616*	*n = 627*	*n = 653*
FCHV	30.7 (189)	35.1 (220)	48.1 (314)
Doctor/nurse/ANM	89.4 (551)	90.0 (564)	95.3 (622)
TBA/AHW/HA/other	21.3 (131)	23.6 (148)	5.4 (35)
Used iron/folic acid tablets during pregnancy	84.4 (582)	89.9 (620)	90.0 (621)
Delivery location			
Home	52.9 (365)	50.6 (349)	33.0 (228)
Health facility	44.9 (310)	48.1 (332)	65.4 (451)
In transit to facility	2.2 (15)	1.3 (9)	1.6 (11)

^a^Response categories were not mutually exclusive

Data are % (n) unless otherwise specified

FCHV denotes Female Community Health Volunteer; *ANM* auxiliary nurse midwife

**Fig. 1:**
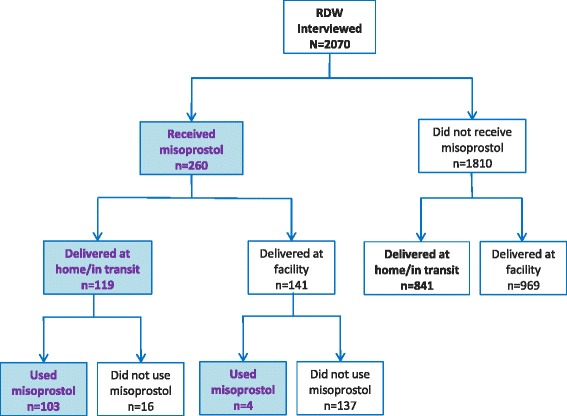
Receipt and use of advance misoprostol, by delivery location

**Fig. 2:**
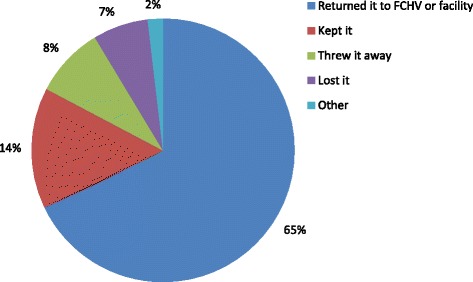
Actions taken with unused misoprostol tablets, as reported by recently delivered women (*n* = 168)

The receipt and use of advance misoprostol for PPH prevention are summarized in Table 2. Among all recently delivered women surveyed, only 15% received information about misoprostol during pregnancy and 13% received misoprostol before giving birth. Among those who received advance misoprostol, 41% used it for PPH prevention; of whom nearly 70% reported correct use, defined as taking all three tablets immediately after delivery of the baby and prior to placental delivery. Among those who reported incorrect use of misoprostol, one woman took the misoprostol during labor but before delivery of the baby, 22 women took the misoprostol after delivery of the placenta, and 11 others took just one or two tablets instead of three. There were no reports of using the misoprostol for labor induction. Figure 1 shows the flow of misoprostol receipt and use by delivery location. Among those who received advance misoprostol and delivered in a facility, use of misoprostol was very low (3%). Among those who received advance misoprostol and gave birth at home, the majority (87%) used the misoprostol for PPH prevention. Among those who received advance misoprostol but did not use it, two-thirds reported that they returned it to an FCHV or health facility, and there were no reports of giving it to someone else or selling it (see Figure 2).

**Table 2:** Recently delivered women: Receipt and use of misoprostol for PPH prevention: % (n)

	Mountain region	Hill region	Terai region	P-value^a^
	(*n* = 690)	(*n* = 690)	(*n* = 690)	
Received information during pregnancy	9.7 (67)	19.3 (133)	15.2 (105)	<0.001
Received misoprostol during pregnancy	7.5 (52)	17.3 (119)	12.9 (89)	<0.001
*Among those who received misoprostol*	*n = 52*	*n = 119*	*n = 89*	
Source of misoprostol				0.005
FCHV	80.8 (42)	78.2 (93)	94.4 (84)	
Health facility provider	17.3 (9)	21.0 (25)	5.6 (5)	
Friend/family	1.9 (1)	0.8 (1)	0.0 (0)	
Used misoprostol for PPH prevention	48.1 (25)	45.4 (54)	31.5 (28)	0.069
*Among those who used misoprostol*	*n = 25*	*n = 54*	*n = 28*	
Used all 3 tablets	84.0 (21)	90.7 (49)	92.9 (26)	0.641
Used immediately after delivery of baby	80.0 (20)	75.9 (41)	64.3 (18)	0.391
Used misoprostol correctly^b^	68.0 (17)	72.2 (39)	64.3 (18)	0.764
Would recommend miso to others (*n* = 98)	100.0 (25)	100.0 (47)	96.2 (25)	0.520

^a^We used Pearson’s Chi-Square and Fisher’s Exact tests (2-sided) to assess the significance of regional differences

^b^Correct use was defined as taking all 3 tablets immediately after delivery of baby and prior to placental delivery

Table 3 summarizes the background characteristics and PPH prevention-related services of the FCHVs. Nearly all reported advance distribution of misoprostol for PPH prevention, the majority during the eighth month of pregnancy as per program standards. About three-quarters counseled clients on the purpose of misoprostol and when to take the tablets, although only one-half reported counseling clients on the number of tablets to take or where to go if PPH occurs. Discussion of the side effects of misoprostol or how to recognize PPH was less common. At the time of interviews, the supply of misoprostol was universally low. Overall 81% of FCHVs reported experiencing one or more stock outs within the past year and 68% had no misoprostol in stock at the time of the interview.

**Table 3:** Female Community Health Volunteers: Background characteristics and PPH prevention services

	Mountain region	Hill region	Terai region
	(*n* = 90)	(*n* = 90)	(*n* = 90)
Background			
Mean age in years ± SD	34 ± 8	41 ± 11	41 ± 9
Educational attainment			
None	23.3 (21)	51.1 (46)	28.9 (26)
Primary	57.8 (52)	45.6 (41)	64.4 (58)
Secondary plus	18.9 (17)	3.3 (3)	6.7 (6)
PPH PREVENTION SERVICES			
Provision of misoprostol	94.4 (85)	98.9 (89)	93.3 (84)
*Among those who provide misoprostol*	*n = 85*	*n = 89*	*n = 84*
Month misoprostol is normally given			
7th	1.2 (1)	1.1 (1)	10.7 (9)
8th	77.7 (66)	78.7(70)	86.9(73)
9th	20.0(17)	13.5(12)	2.4(2)
Not sure	1.2(1)	6.7 (6)	0.0 (0)
Counseling includes information about:			
Purpose of misoprostol	90.6(77)	66.3(59)	78.6(66)
When to take the tablets	78.8 (67)	69.7 (62)	72.6 (61)
Number of tablets to take	57.6 (49)	53.9 (48)	54.8 (46)
Possible side effects	17.6 (15)	23.6 (21)	34.5 (29)
How to recognize PPH	10.6 (9)	31.5 (28)	27.4 (23)
Where to go/what to do if PPH occurs	48.2 (41)	39.3 (35)	63.1 (53)
Receipt of supervision within last 12 months			
Yes	61.1 (55)	25.6 (23)	43.3 (39)
No	38.9 (35)	74.4 (67)	56.7 (51)
Receipt of supervision on misoprostol for PPH prevention			
Yes	18.0 (16)	25.6 (23)	32.2 (29)
No	82.0 (73)	74.4 (67)	67. 8 (61)
MISOPROSTOL STOCK			
# of tablets currently in stock			
None	67.1 (57)	65.2 (58)	71.1 (59)
1–6	18.8 (16)	22.5 (20)	21.7 (18)
7–12	8.2 (7)	10.1 (9)	6.0 (5)
> 12	5.9 (5)	2.3 (2)	1.2 (1)
Any stock out within the last year	87.1 (74)	69.7 (62)	86.9 (73)
*Among those with any stock out*	*n = 74*	*n = 62*	*n = 73*
Mean duration of stock out in months ± SD	8.2 ± 4.3	5.1 ± 4.5	6.3 ± 4.7
Actions taken at time of stock out^a^			
Referred to health facility	70.3 (52)	62.9 (39)	74.0 (54)
Referred to private clinic	0.0 (0)	3.2 (2)	1.4 (1)
Borrowed tablets from other FCHV	0.0 (0)	0.0 (0)	2.7 (2)

^a^Response categories were not mutually exclusive

Data are n (%) unless otherwise specified. Percentages have been rounded

FCHV denotes Female Community Health Volunteer; *SD* standard deviation

The characteristics and PPH-related services of the 99 health facilities included in the study are presented in Table 4. About two-thirds were sub-district level birthing centres, which are increasingly responsible for deliveries in the rural areas of Nepal. Among those providing obstetric services, the provision of oxytocin for PPH prevention was universal. Nearly one-quarter of facilities also reported the provision of misoprostol for PPH prevention. Oxytocin stock was considered to be sufficient at nearly all facilities with exception of a few in the mountain and hill regions. In contrast, only about one-half of facilities had sufficient stock of misoprostol at the time of this assessment.

**Table 4:** Health facility characteristics and PPH prevention services

	Mountain region	Hill region	Terai region
	(*n* = 33)	(*n* = 33)	(*n* = 33)
Facility level			
District hospital or above	9.1 (3)	12.1 (4)	9.1 (3)
Primary	6.1 (2)	9.1 (3)	9.1 (3)
Health post	45.5 (15)	21.2 (7)	33.3 (11)
Sub-health post	39.4 (13)	57.6 (19)	48.5 (16)
Facility type			
CEONC	0.0 (0)	6.1 (2)	6.1 (2)
BEONC	9.1 (3)	6.1 (2)	6.1 (2)
Birthing centre	54.6 (18)	63.6 (21)	75.8 (25)
Non-birthing centre	36.4 (12)	24.2 (8)	12.1 (4)
*Among facilities offering obstetric services* ^a^	21	25	29
AMTSL interventions provided:			
Uterotonics	100.0 (21)	100.0 (25)	100.0 (29)
Controlled cord traction	98.7 (20)	100.0 (25)	100.0 (29)
Uterine massage	98.7 (20)	100.0 (25)	100.0 (29)
Type of uterotonics used for PPH prevention^b^			
Oxytocin	100.0 (75)	100.0 (75)	100.0 (75)
Misoprostol	19.1 (4)	56.0 (14)	10.3 (3)
Methergine/ergometrine	23.8 (5)	32.0 (8)	17.2 (5)
Uterotonics currently in stock at facility			
Oxytocin	95.2 (20)	100.0 (25)	100.0 (29)
Misoprostol	85.7 (18)	80.0 (20)	58.6 (17)
Methergine/ergometrine	19.1 (4)	16.0 (4)	20.7 (6)
Current stock of uterotonic is sufficient			
Oxytocin	85.7 (18)	96.0 (24)	100.0 (29)
Misoprostol	57.1 (12)	56.0 (14)	41.4 (12)

^a^This included all facilities in the sample except the non-birthing centres, which were not equipped to offer uterotonics during delivery at the time of this evaluation

^b^Response categories were not mutually exclusive

## Discussion

This assessment identified widespread gaps in the supply of misoprostol among FCHVs and within health facilities in all three ecological regions. Given the central importance of misoprostol to the advance distribution program, irregular and/or insufficient supply is a key barrier to program success. Efforts to identify and address logistical and other supply chain constraints are therefore needed as is more consistent and up-to-date monitoring of misoprostol stock throughout the health system. Since birthing centres are primarily responsible for deliveries in rural areas, priority should be given to ensuring sufficient misoprostol supply at these and other sub-district level facilities. In response to preliminary study findings, the government has initiated efforts to identify and address the sources of the misoprostol supply shortages. As an example, the government’s Logistics Management Division is currently piloting a new electronic system that will enable facility-based tracking of health supplies, including misoprostol, at the district level and above. The government has also continued to expand the advance distribution program, which has now been implemented in 42 out of 75 districts country-wide.

Lessons could be learned from the success of the oxytocin component of the PPH Prevention Program in Nepal. In this study, there was a sufficient supply of oxytocin at nearly all health facilities that provide obstetric care. Implementation of the oxytocin program has been underway for many years and has been a collaborative effort of the FHD and various multi-lateral and non-governmental organizations. These are the same entities involved in the advance distribution program and, as a result, comparison of key similarities and differences in program implementation strategies and supply management procedures may be helpful. The program may also benefit from a change made in 2014, in which the distribution of misoprostol was combined with distribution of chlorhexidine for cord care and birth preparedness packages (BPP). All three technologies are now provided by FCHVs in the eighth month of pregnancy, thus potentially boosting commitment to the advance distribution program among FCHVs.

Study findings also suggest a need for improving knowledge of and commitment to the advance distribution program among FCHVs. Discussion with FCHVs after completion of this evaluation revealed confusion about the importance of the advance distribution program given the government’s emphasis on facility births. As part of the push to promote facility births, FCHVs formerly received financial incentives for ensuring that women in their communities delivered in facilities, Hence, at the time of this evaluation, many were likely unaware of the continuing need to provide advance misoprostol. In light of this situation, the importance of the advance distribution program should be stressed to all FCHVs, even when women indicate a preference for facility deliveries. The study findings also revealed some confusion about when to provide advance misoprostol (16% were providing it in the seventh or ninth month instead of the eighth month). In addition, many FCHVs were not providing full information about when and how to use misoprostol, or how to recognize PPH. Refresher trainings or review meetings that emphasize proper counseling and misoprostol provision would likely help. In response to initial information about these findings, the government has also increased its emphasis on refresher trainings for FCHVs.

Another option being considered by the Ministry of Health and Population (MoHP) is the replacement of FCHVs with a new cadre of community providers with more training—auxiliary nurse midwives (ANMs) who could provide antenatal care as well as assist deliveries both in the home and in health facilities. This would likely be more costly and time consuming, however, and following an initial pilot project involving community ANMs in one district, there has been no subsequent effort to expand this idea.

The high rate of institutional delivery, which was 54% overall and 65% in the terai region, is encouraging. This reflects considerable change in a very short period of time, from an estimated 35% of deliveries in the 2011 DHS survey [2]. There has been some concern that advance distribution of misoprostol would deter women from going to facilities, but these data suggest that has not been the case. The findings should also alleviate concerns about diversion of misoprostol for uses other than PPH prevention. There was no evidence that misoprostol was used for any other purpose (including labor induction and abortion). The majority of those who did not use their advance misoprostol returned it after the birth and most others either threw it away or kept it. Another recent study of community-based distribution of misoprostol for PPH prevention obtained similar results [12].

The high use of misoprostol (87%) among those who received it and delivered at home is also encouraging and mirrors findings from other similar studies conducted in Ethiopia, Ghana, Liberia and South Sudan [12–15]. Furthermore, most women reported correct use of misoprostol, underscoring that when given accurate information, women can use misoprostol properly on their own. There is no published data, however, on whether the successful implementation of advance distribution programs in other countries was sustained over time or as the programs were scaled-up. The evaluations published thus far are largely reports from pilot programs or the period during or just after initial program implementation.

Nepal was one of the first countries to implement a community-based advance distribution program and at the time of this assessment, had been involved in scaling the program up for about 5 years. While it is encouraging that the government remains committed to the PPH prevention program and is working to address the gaps identified in this study, future research should examine more closely whether these gaps have been sufficiently addressed as well as the costs and benefits of long-term investment in a program that may only reach a small share of the target population. Information about the long-term status of similar programs in other countries would also be of interest. While the clinical benefits of advance distribution of misoprostol for home births have been established [16] and women appear willing and able to use misoprostol properly, questions remain about the scalability and magnitude of impact of such programs over the long-term.

### Strengths and limitations

The mixed methods approach used in this evaluation enabled assessment of multiple aspects of the PPH prevention program. In addition, the large number of women included in the household surveys and the large number of facilities and FCHVs surveyed enabled us to assess and compare the program status in all three of the country’s ecological regions. However, the responses obtained in the household interviews may have been subject to some recall bias, particularly among those who delivered closer to 1 year prior to the date of interview.

## Conclusions

The findings from this study confirm prior research indicating that women who receive advance misoprostol in the third trimester of pregnancy are largely willing and able to use it correctly for PPH prevention during home-births. At the same time, the supply and personnel challenges identified are key barriers to program success and raise questions about the scalability and magnitude of impact of the advance distribution program over the long-term.

## Abbreviations

ANM: Auxiliary nurse midwife
BPP: Birth preparedness package
FCHV: Female Community Health Volunteer
FHD: Family Health Division
MoHP: Ministry of Health and Population
MSC: Matri Surakhchya Chakki
PPH: Postpartum hemorrhage
